# A qualitative study exploring the barriers and facilitators of implementing a cardiovascular disease risk reducing intervention for people with severe mental illness into primary care contexts across England: the ‘PRIMROSE’ trial

**DOI:** 10.1186/s12913-020-05643-2

**Published:** 2020-08-15

**Authors:** Suzan Hassan, Samira Heinkel, Alexandra Burton, Ruth Blackburn, Tayla McCloud, Jamie Ross, David Osborn, Kate Walters

**Affiliations:** 1 Research Department of Primary Care and Population Health, University College London, Upper Third Floor, UCL Medical School (Royal Free Campus), Rowland Hill Street, London, NW3 2PF UK; 2 Division of Psychiatry, University College London, 6th Floor, Wing B, Maple House, 149 Tottenham Court Road, London, W1T 7NF UK; 3 Camden and Islington NHS Foundation Trust, 4 St Pancras Way, London, NW1 0PE UK

**Keywords:** Severe mental illness, Physical health, Cardiovascular disease, Primary care, Nurse, Normalisation process theory, Implementation science, Qualitative, Barriers, Facilitators

## Abstract

**Background:**

People with severe mental illness (SMI) are at greater risk of earlier mortality due to physical health problems including cardiovascular disease (CVD). There is limited work exploring whether physical health interventions for people with SMI can be embedded and/or adopted within specific healthcare settings. This information is necessary to optimise the development of services and interventions within healthcare settings. This study explores the barriers and facilitators of implementing a nurse-delivered intervention (‘PRIMROSE’) designed to reduce CVD risk in people with SMI in primary care, using Normalisation Process Theory (NPT), a theory that explains the dynamics of embedding or ‘normalising’ a complex intervention within healthcare settings.

**Methods:**

Semi-structured interviews were conducted between April–December 2016 with patients with SMI at risk of CVD who received the PRIMROSE intervention, and practice nurses and healthcare assistants who delivered it in primary care in England. Interviews were audio recorded, transcribed and analysed using thematic analysis. Emergent themes were then mapped on to constructs of NPT.

**Results:**

Fifteen patients and 15 staff participated. The implementation of PRIMROSE was affected by the following as categorised by the NPT domains: 1) Coherence, where both staff and patients expressed an understanding of the purpose and value of the intervention, 2) Cognitive participation, including mental health stigma and staff perceptions of the compatibility of the intervention to primary care contexts, 3) Collective action, including 3.1. Interactional workability in terms of lack of patient engagement despite flexible appointment scheduling. The structured nature of the intervention and the need for additional nurse time were considered barriers, 3.2. Relational integration i.e. whereby positive relationships between staff and patients facilitated implementation, and access to ‘in-house’ staff support was considered important, 3.3. Skill-set workability in terms of staff skills, knowledge and training facilitated implementation, 3.4. Contextual integration regarding the accessibility of resources sometimes prevented collective action. 4) Reflexive monitoring, where the staff commonly appraised the intervention by suggesting designated timeslots and technology may improve the intervention.

**Conclusions:**

Future interventions for physical health in people with SMI could consider the following items to improve implementation: 1) training for practitioners in CVD risk prevention to increase practitioners knowledge of physical interventions 2) training in SMI to increase practitioner confidence to engage with people with SMI and reduce mental health stigma and 3) access to resources including specialist services, additional staff and time. Access to specialist behaviour change services may be beneficial for patients with specific health goals. Additional staff to support workload and share knowledge may also be valuable. More time for appointments with people with SMI may allow practitioners to better meet patient needs.

## Background

It is well established that people with a diagnosis of severe mental illness (SMI) are at greater risk of early mortality compared to the general population due to physical health problems. One of the physical health problems that people with SMI experience includes cardiovascular disease (CVD) [1, 2], and the mortality gap between people with SMI and the general population is widening [3]. The causes of this health disparity are multi-factorial and interrelated [2]. Higher cholesterol, blood pressure, blood glucose and obesity are apparent in people with SMI as well as unhealthy behaviours such as smoking, excessive alcohol intake, poor diet and physical inactivity [2]. Restricted access to appropriate healthcare has also been reported as a potential contributing factor, with barriers to access including difficulties around patients attending appointments, knowledge, stigma, lack of interpersonal skills displayed by healthcare professionals (HCPs) and lack of continuity in HCPs where patients see different HCPs for their care rather than the same HCPs [4–7]. Current clinical guidance in the United Kingdom (UK) states that both primary and secondary care services should take a more active role in detecting and preventing health problems in people with SMI [8].

There is an emerging body of research investigating the effects of different interventions to reduce physical health problems in people with SMI. Systematic reviews have reported nutrition interventions to be effective at preventing and treating weight gain, bupropion and varenicline to be effective at improving smoking quit rates in the medium and long term, and inconsistencies in the literature on interventions aimed at improving sedentary behaviour and physical activity levels [9–11]. It is however unclear whether these interventions are replicable, can be embedded and/or adopted within different healthcare contexts and whether factors related to setting may impact the effectiveness of interventions.

There is limited existing research on factors that affect the delivery of physical healthcare for people with SMI in healthcare settings overall and in particular primary care. Two studies conducted in Australia; one in both community mental health settings and primary care settings and the other only in community mental health settings [12, 13]. These studies reported that the availability of services, geographic location, waiting time, staffing levels, mental health stigma amongst staff, lack of role responsibility, lack of training, lack of primary care links and increased workload all affect delivery [12, 13]. Research in the UK with mental and physical health staff has reported that having appropriate mental and physical health knowledge and skills amongst staff, prioritising physical health, information sharing systems, access to time and shared agreement of roles and responsibilities were important [14]. Factors preventing healthcare delivery from the perspectives of both primary care and community mental health professionals included challenges accessing General Practitioner (GP) and community-based services, challenges adopting healthy behaviours, patients not attending appointments and a lack of awareness among HCPs of CVD risk in people with SMI [15]. We identified no studies that explored factors affecting delivery of physical health care to people with SMI in primary care settings alone.

We developed a pragmatic behavioural intervention (PRIMROSE) delivered by primary care nurses and healthcare assistants (HCAs) in primary care practices across England to people with SMI, to reduce their CVD risk [16, 17]. In UK primary care settings, 98% of the UK population is registered with a GP. In this setting nurses and HCAs provide a range of care to registered patients including, for example, routine health checks, screening services, reviews of long-term conditions (e.g. diabetes, pre-diabetes, asthma), immunisations, wound care, contraceptive services, weight management etc. They would not normally provide specific care for people with SMI, but might provide care for other health conditions for people with SMI. To our knowledge, in current primary care contexts in the UK there are no interventions where a manual is provided to guide primary HCPs to target CVD risk in people with SMI. The PRIMROSE intervention comprised of behaviour change components designed to improve health behaviours such-as physical activity, diet, alcohol use and smoking in people with SMI and encourage the uptake of physical health medications such-as statins to reduce CVD risk in people with SMI. This is described further in the supplementary material. The findings from the cluster randomised trial of the PRIMROSE intervention in 76 practices has been published [16, 17]. There was no effect on the primary outcome (total cholesterol) or other secondary outcomes compared to usual treatment. However, it was associated with fewer costs due to reduced psychiatric admissions. It is unclear whether this was a result of the problems related to the implementation of the intervention into primary care. It is therefore important to understand factors that impeded and/or assisted intervention implementation into primary care contexts to further elucidate findings, as well as further inform the implementation of other physical health interventions more broadly in primary care other than CVD risk reducing interventions alone. Research in this area is limited and more evidence is needed to help inform future services, clinical guidelines and commissioning groups working to implement physical health interventions for people with SMI in primary care settings.

Qualitative methods are advocated to explore the implementation of complex interventions [18]. Additionally, the use of theory is thought to strengthen knowledge and explanations regarding why interventions may or may not work well within specific contexts [18, 19]. One theory that explains the dynamics of embedding or ‘normalising’ a complex intervention within settings is Normalisation Process Theory (NPT) [20, 21]. NPT was developed to address the difficulties of implementing new interventions and/or care into healthcare settings, and to provide greater explanation behind these processes. NPT has been applied widely in different populations and healthcare settings, but not yet to a physical health primary care intervention for people with SMI.. The purpose of the current study was to explore the barriers and facilitators of implementing the PRIMROSE intervention into primary care across England, applying NPT to facilitate a deeper understanding of the factors that affected implementation.

## Methods

### Participants and recruitment

Patients were eligible for inclusion for the PRIMROSE study if they were aged 30–75 years old, on the GP practice mental health register with a diagnosis of SMI (schizophrenia, persistent delusional disorder, schizoaffective disorder, bipolar disorder, psychosis, psychotic depression or other psychotic disorder), a total cholesterol level above or including 5.0 mmol/l or raised total cholesterol/ HDL cholesterol ratio above and including 4 and one or more of the following: BMI ≥ 30 kg/m2, current smoker, blood pressure ≥ 140 mmHg systolic and/or ≥ 90 mmHg diastolic, HbA1c of 42 to 47 mmol/mol (6.0 to 6.4%) and/or impaired fasting glucose (5.5 to 6.9 mmol/L), diagnosis of diabetes, diagnosis of hypertension. Staff delivering the intervention had to be working within a recruited GP practice as either a nurse or HCA [17].

A random 20% sample of practices randomised to deliver the PRIMROSE intervention in the trial were identified (*n* = 8/38); from which thirty patients receiving the PRIMROSE intervention were invited to participate. Staff were selected if they had delivered at least one PRIMROSE session, attended PRIMROSE training and were not part of the internal PRIMROSE pilot phase (*n* = 31/41 health providers). Staff and patients were approached for participation by researchers (S.H2 and A.B) via email and letter. Ethical approval for the study was granted by the London - City Road & Hampstead Research Ethics Committee NRES committee, REC ref. 12/LO/1934.

### Data collection

Face-to-face semi-structured interviews were conducted with participants by two members of the research team (S.H2 and A.B). None of the researchers had any prior contact with patients. There was prior contact between researchers and staff in terms of training and answering queries or concerns related to the PRIMROSE trial.

Staff interviews took place between April – August 2016, approximately 6–18 months after conducting the final appointment with their last PRIMROSE patient. Patient interviews took place between October – December 2016, approximately 6–9 months after their final intervention appointment. Interviews took place in primary care practices.

Two topic guides aimed at patients and staff were used to guide interviews (see supplementary material). These contained open-ended questions on the impact of the intervention on patients, benefits and disadvantages of the intervention, fitting the intervention into current roles and primary care contexts and factors impacting this. Corresponding prompts were added to questions to gain more clarity or detail regarding responses.

Questions in staff and patient topic guides were developed through discussion with the core research team (S.H2, A.B, K.W and D.O). The topic guide was piloted on members of the research team and an HCP to check the relevance of topics and legibility of questions and adapted accordingly.

Before the start of the interview, researchers explained the purpose of the study and encouraged participants to share both negative and positive experiences of receiving and delivering the PRIMROSE intervention. Staff were required to complete a form to ascertain demographic details, whilst patient characteristics were collected during the PRIMROSE trial [16, 17].

All interviews were audio-recorded and transcribed verbatim by an external transcription company. The transcripts were checked against audio-recordings for accuracy by the researchers. Researchers anonymised transcripts by removing all identifiable content.

### Data analysis

Anonymised and corrected transcripts were stored and analysed on NVivo (Version 11) software. The analysis was conducted by researchers with a background in qualitative research, health psychology, psychiatry and mental health research. In the initial analysis researchers (S.H2, A.B, R.B and T.M) familiarised themselves with the data by reading the transcripts and then coded the data descriptively to represent emerging topics. The codes were developed through discussion with the research team (S.H2, A.B, R.B, T.M, D.O and K.W). This process was iterative and researchers continuously revised and adapted codes until they felt satisfied that the codes represented the data. Once coded, the data were then analysed thematically [22] by S.H2 and A.B. Two further researchers (S.H1 & J.R) with an interest in mental health research, process evaluations and implementation science were involved in further developing the themes. These researchers had previously not been involved with the trial or data collection. Themes were identified inductively by searching for commonalities, discordant views and underlying meanings behind the derived codes. The themes were derived iteratively through discussion with the research team (S.H1, J.R, A.B, S.H2, K.W and D.O).

Following agreement on themes, NPT was applied to move the analysis beyond description and toward explanation. The four main constructs include coherence (i.e. sense-making of the intervention), cognitive participation (i.e. commitment to and engagement with intervention), collective action (i.e. the work that is conducted to facilitate delivery) and reflexive monitoring (i.e. an evaluation of the costs and benefits). Each construct contains sub-components; however, we were particularly interested in the sub-components of collective action, including: interactional workability, contextual integration, skill-set workability and relational integration as this is one of the most defined and used of the subconstructs and particularly helpful in explaining the work that is done around intervention implementation [20, 21]. Further, the sub-constructs related to other domains of the NPT appeared to overlap and it was not possible to map themes to these sub-constructs without repeating themes in multiple areas. However, when mapping the themes to sub-constructs of collective action, there was greater distinction between the themes and sub-components that they were mapped to. One researcher (S.H1) mapped the inductively derived themes to NPT constructs. Details regarding how the NPT constructs were operationalised are provided in Table 1. The mapping process was iterative, moving backward and forward between the emergent themes and the NPT definitions. The mapping process was discussed among researchers (S.H1, J.R, A.B and K.W) and revised iteratively until we were satisfied that the themes had been mapped correctly onto the NPT constructs.

**Table 1:** Operationalisation of Normalisation Process Theory

NPT constructs	Operationalisation of constructs^a^
Coherence	Whether patients and staff were able to understand the purpose of the intervention. Exploring participants views on the meaning of the intervention including whether staff and patients perceived the intervention as beneficial in terms of reducing health problems.
Cognitive participation	Whether patients and staff were prepared and willing to commit to and engage with the intervention.
Collective action	Establishing what work was carried out in terms of interactional workability, relational integration, skill-set workability and contextual integration in order to facilitate delivery. This is further explained below.
*Interactional workability*	How staff encouraged patient interaction with the intervention in the context of primary care practices in terms of accessibility and flexibility of delivery.
*Relational integration*	How the work that was done to facilitate the delivery of the intervention was understood across staff within practices (even if they were not responsible for delivering the intervention) and whether there was cohesion between staff and patients.
*Skill-set workability*	Whether staff and patients perceived that staff possessed the skills, training and knowledge to deliver the intervention.
*Contextual integration*	The practices’ ability to support the intervention as well as the fit of the intervention into practice contexts.
Reflexive monitoring	Whether staff and patients evaluated ways to adapt intervention.

^a^NPT constructs were operationalised according to NPT construct definitions [20, 21]

### Study integrity

We ensured that various steps were taken to maximise the integrity of the study findings. Two researchers (A.B & S.H2) were involved in collecting the data which allowed them to engage and observe participants and their responses. These researchers were also involved in the analysis process and their familiarity with the data allowed the wider team to interpret data in the context in which it was collected. We also collected data from two sources, including patients and staff. We triangulated patient and staff responses, which allowed us to compare both perspectives, thereby gaining a comprehensive picture of the implementation of the intervention overall. We also discussed the coding strategy, development of themes and mapping of NPT between ourselves. As discussed previously, we have a multidisciplinary background with specialisms in different subject areas. This allowed us to consider the interpretation of data in various ways. We also actively identified both common atypical themes as a way of ensuring as much as possible that different perspectives were taken into consideration.

## Results

### Participant characteristics

Thirty participants, including 15 nurses and HCAs who delivered PRIMROSE (from 31 approached) and 15 patients with SMI who received it (from 30 approached), took part in the current study. Six patients did not give a reason for not wanting to take part, however other reasons included inability to gain contact (*n* = 3) and not feeling well enough to take part (*n* = 2). Nine staff did not respond to the invitation to take part and reasons for non-participation included: not interested (*n* = 3), lack of time (*n* = 2) and not yet finished delivering the intervention (*n* = 2).

The characteristics of staff are presented in Table 2 compared to the characteristics of the staff delivering PRIMROSE. The sample comprised of both practice nurses and HCAs of different age ranges (25–65 years) with varying degrees of professional experience (from 1 to 30 years). Staff were all White British ethnicity and female. Most staff were previously not involved in research. The sample in the present study were mostly comparable to the rest of the staff delivering PRIMROSE in terms of age, length of experience and previous research experience, but did not capture the perspectives of the few ethnic minority groups, male participants, those delivering 0–1 or 26–35 PRIMROSE intervention appointments or the only GP delivering PRIMROSE.

**Table 2:** Staff characteristics in the qualitative sample compared to the characteristics of staff delivering PRIMROSE

Characteristics	Qualitative Staff sample (*n* = 15)	All PRIMROSE Staff (*n* = 41)
Age group		
< 25	–	1
25–35	3	8
36–45	1	9
46–55	7	15
56–65	4	8
Gender		
Female	15	39
Male	–	2
Ethnicity		
White British	15	36
White Other	–	3
Asian Other	–	2
Provider role		
Healthcare Assistant	6	22
Practice Nurse	7	15
Research Nurse	2	3
GP	–	1
Length of experience as a nurse/HCA (years, months)		
< 1 year	–	1
1 to 2 years	1	4
3 to 5 years	3	9
6 to 10 years	4	10
11 to 15	2	5
16 to 20	1	6
21 to 30	4	5
Unknown	–	1
Previous experience of research		
Yes	6	16
No	9	25
Number of PRIMROSE intervention appointments delivered		
0–1	–	2
2–5	1	3
6–10	1	6
11–15	2	5
16–20	2	8
21–25	3	4
26–30	–	1
31–35	–	2
36–40	1	1
41–45	3	4
46–50	1	1
51–55	1	2

The characteristics of the patients in the present study are listed in Table 3 compared to the characteristics of the rest of the PRIMROSE trial participants. The sample in the present study comprised of participants who were diagnosed with either bipolar disorder or schizophrenia. The age of patients ranged from 30 to 70 years. Patients were mostly male and White ethnicity. The characteristics of the patient sample were similar to those in the overall PRIMROSE trial sample in terms of gender, age group and diagnosis. However, the sample in the present study did not capture the perspectives of the few ethnic minority groups in PRIMROSE, the minority of those that were separated, divorced or widowed as well as those that received either 0 or 1 PRIMROSE intervention appointment.

**Table 3:** Patient characteristics compared to the characteristics of the patient intervention sample

Characteristics	Qualitative patient Sample (*n* = 15)	All patients allocated to the PRIMROSE intervention (*n* = 155)
Gender		
Male	9	67
Female	6	88
Age group		
30–39	2	25
40–49	4	48
50–59	3	38
60–69	4	31
70+	2	12
Marital Status		
Single	8	66
Married or cohabiting	7	59
Separated or divorced	–	25
Widowed	–	4
Ethnicity		
White	13	134
Black	–	11
Asian	2	5
Other	–	4
Diagnosis		
Schizophrenia/ schizo-affective disorder	6	54
Bipolar affective disorder	7	71
Other Psychosis	2	30
Number of PRIMROSE intervention appointments attended (over 6 months)		
0	–	32
1	–	15
2–5	3	36
6+	12	72

### Findings

The themes derived from the data are provided in Table 4. These are presented alongside the NPT constructs that themes were mapped to. The themes were discussed with specific reference to, and organised by, the NPT constructs in the written presentation of findings. Some themes mapped on to more than one NPT construct. In these instances, the relevance of the theme regarding different NPT constructs were discussed within each NPT heading. When comparing themes across staff and patients, it was apparent that in some cases themes arose in both staff and patient interviews but in other cases may have arisen only in staff or patient interviews.

**Table 4:** Inductively derived themes mapped to NPT constructs

Themes identified in the raw data	Broad theme/NPT constructs
Clarity of purpose	Coherence
Value of intervention	Coherence
Mental health stigma	Cognitive participation
Confidence to engage	Skill-set workability (Collective action)
Motivation to engage	Cognitive participation
Compatibility with existing practice	Cognitive participation Interactional workability
Accessibility of intervention	Interactional workability
Engagement with intervention	Interactional workability (Collective action)
Intervention materials	Interactional workability (Collective action
Resource availability and benefits	Contextual integration (Collective action) Reflexive monitoring
The level of ‘in-house’ support	Relational integration (Collective action)
Patient – staff alliance	Relational integration (Collective action)
Knowledge	Skill-set workability (Collective action)
Training	Skill-set workability (Collective action)
Skills	Skill-set workability (Collective action)
Modifiability through accessibility	Reflexive monitoring

### Coherence

The intervention was mostly perceived by patients as coherent in terms of the aim of the intervention. Both staff and patients reported a shared understanding of the benefits of the intervention.

#### Clarity of purpose

A common theme among patients was a clear understanding of the purpose of the intervention, acknowledging the focus on health improvement in people with SMI to reduce CVD risk. Patients reported that their understanding was facilitated by staff who provided relevant information sheets and explained the purpose.

*“…I thought it was to get an insight into how I was going from time to time, seeing people you know, getting weighed, taking blood pressure and things like that …I got the original pamphlet and I read that and that kind of told me everything I wanted to know.”* (Patient 12, female, 70’s)

An atypical view among patients was confusion regarding the purpose of the intervention. One patient believed that the intervention was designed to improve mental health outcomes rather than physical health. The lack of understanding appeared to be caused by the GP’s description of the intervention.

*“As I understood it, it was basically, with early intervention, or regular intervention, by your local GP practice, the nurse, normally, then it can offer stability and assistance so people like myself don’t relapse... I was approached by the GP practice, basically saying, would I be happy to take part in a project related to mental health.”* (Patient 81, male, 60’s)

#### Value of intervention

The intervention was perceived as valuable by patients and staff. Staff reported that they understood the intervention could prevent patients from experiencing later health problems and increase quality of life, as well as reduce financial burden for future health services.

*“I think it would benefit people. Because it’s a positive thing, and it’s working towards improving people’s health and their lifestyles.”* (Staff 8, Nurse, 50’s)

A common theme among patients was that the intervention would provide an opportunity to make changes and improvements to their health.

*“I thought it would be a good idea to just look at my healthcare and try and make some necessary adjustments so that my health can be improved…”* (Patient 112, female, 50’s)

### Cognitive participation

Although most staff and patients understood the purpose and value of the intervention (i.e. coherence), the extent to which staff were cognitively willing to participate, engage and commit to the intervention varied. Mental health stigma in some cases resulted in negative perceptions among staff of their ability to implement the intervention. In some cases, an understanding (i.e. coherence) of the value of the intervention in terms of helping patients become healthier, motivated staff to deliver the intervention. In other cases, difficulties arising from the contextual environment (collective action/contextual integration) affected cognitive participation.

#### Mental health stigma

Staff held different views regarding their preconceptions of mental health. There were some prior concerns regarding working with people with SMI. Some staff anticipated problems around the impact of mental health symptoms on attendance and engagement difficulties.

*“We can deal with somebody with diabetes and all of that, and we can tell them this, that and the other, but somebody with mental health, when they’ve got that problem they may not have that understanding. They may not engage for a long period of time. It’s really very difficult… I prefer people who can engage with me.”* (Staff 5, Nurse, 40’s)

However, other staff felt positive about working with patients with SMI and in some cases prior experience within nursing roles enabled staff to feel open toward delivering the intervention.

*“I think you have to be open-minded, as a nurse, to be a good nurse. So I wasn’t intimidated at all, initially”* (Staff 4, HCA, 50’s)

#### Motivation to engage

Despite some negative attitudes towards mental illness in some individuals, it was clear that most staff had the motivation and desire to help patients achieve their goals and therefore engage with the intervention. This appeared to stem from the understanding of its purpose and potential benefits (i.e. coherence).

*“I think probably knowing that you could be a part of helping them, I think that probably influenced us as well, and knowing that if you just gave them that little bit of help then they could improve. I think that’s probably the motivation in that.”* (Staff 15, Nurse, 60’s)

#### Compatibility with existing practice

Some issues related to cognitive participation were also underpinned by difficulties related to contextual integration (discussed later). Some staff questioned the applicability of the intervention to real-world contexts (cognitive participation) and suggested that the intervention would not fit in within a busy GP practice which subsequently affected their willingness to deliver the intervention going forward.

*“I’m not sure how it would fit in easily in a surgery that’s already quite packed. We’ve got ever-growing lists, so whether it could be done in more of a mental health environment, it may be more appropriate…”* (Staff 13, Nurse, 30’s)

### Collective action

Several barriers surrounding the work that was needed to facilitate delivery of the intervention were identified. There were some problems related to interactional workability, contextual integration, skill-set workability and, in some cases, relational integration.

#### Interactional workability

Staff made substantial efforts to encourage patient engagement by facilitating accessibility to the intervention. This finding was also reported by patients, and staff arranged appointments to suit their preferences. However, staff faced barriers regarding patient engagement. Additionally, both staff and patients found some of the intervention written materials including the use of written health plans, difficult and time-consuming to implement. However, patients expressed mixed views about the use of health plans. It was also difficult to operationalise the intervention into routine practice due to the need for adequate time to facilitate engagement and accessibility.

##### Accessibility of intervention

Staff acknowledged that intervention appointments would sometimes take longer than the time they had available. Difficulties were centred mainly on fitting appointments around additional responsibilities. Despite these difficulties however, staff demonstrated flexibility and scheduled appointments accordingly to increase accessibility.

*“Something, finding slots when we’re so busy, that, that would be a thing as well, so sometimes you think to yourself, well, you know, this patient needs extra time, but actually we haven’t got a slot…to fit her in”* (Staff 1, HCA, 40’s)

The flexibility in scheduling appointments was also reported by patients who suggested that staff would arrange appointments when it would suit them and were understanding even if patients did not attend.

*“She suggested dates on the telephone, and sometimes I would phone and say, I’m not available on that day, so an alternative appointment was made, so it had to be mutually convenient for both of us. Obviously, she has other jobs... in the surgery to do as well, so it had to be convenient for her as well.”* (Patient 112, female, 50’s)

##### Engagement with intervention

Despite attempts to make the intervention accessible to patients through flexible appointment scheduling, staff reported difficulties related to patient attendance. Staff commonly reported being disappointed when patients were disengaged from the intervention given the time invested to facilitate accessibility.

*“…it was the patients that didn’t come. You just get frustrated; you put all of this time and effort into the first appointment and then you never saw them again.”* (Staff 10, Nurse, 40’s)

##### Intervention materials

Staff reported that the materials designed for the intervention including the health plans, in some cases acted as barriers to providing the intervention. Some staff reported that patients sometimes struggled in terms of understanding and completing the required documentation and reported it was time-consuming and negatively impacted on the consultation process.

*“I think using the book for something like that, you do need a lot of time to go through it with them…I think maybe the book made it feel too formulized… I don’t find that book a very easy layout so I think that was almost a stumbling block. Maybe I didn’t understand the book particularly and the patients didn’t particularly find it helpful*” (Staff 11, Nurse, 60’s)

Patients’ views regarding the value of health plans were mixed. Some patients reported that they were sometimes problematic to use in practice. It was suggested that documents were repetitive and were sometimes difficult to fill in. Conversely, others reported that the health plans helped them keep track of the changes they had made.

*“That was okay. A bit repetitive at times because, you know, you were... obviously, the food for four weeks, you tended to be writing a little bit of the same thing…”* (Patient 9, female, 50’s)

*“... That booklet or a personal diary would help, because sometimes you can’t remember exactly what have you done a week ago, two weeks ago, so it’s good to write down some notes. But it’s difficult sometimes. One of the difficulties, I found it’s difficult to do it on the day sometimes.”* (Patient 112, female, 50’s)

##### Compatibility with existing practice

Staff were concerned about patient engagement issues with intervention appointment attendance. As a result, they suggested that the practicalities of getting patients with SMI to engage within a GP practice would be difficult. They also suggested there would be a need for additional nurse time to facilitate engagement and accessibility. However, the availability of additional nurse time was questionable.

*“I think if we were going to deliver that care in that format and at that intensity, I think it would be quite difficult. Not so much the face-to-face time, but certainly the getting people in... Ringing them once wasn’t a problem, just ringing them again and again…if that was going to be part of how we would deliver the care, that could provide difficulties if it was down to me”* (Staff 10, Nurse, 40’s)

The difference between GP and intervention appointments was further highlighted by patients. Patients expressed that more time was available in intervention appointments with care that felt holistic compared to GP appointments. However, this perception may have been influenced by the fact that patients were aware that the care they were receiving were part of an intervention and not usual care.

*“This was totally different. This is very patient-centred… From my view is that it’s very much based on a holistic approach of the patient. So it’s patient-centric and in looking at everything whereas a normal GP appointment is five minutes and it’s transactional…”* (Patient 17, male, 40’s)

One staff member questioned the structured nature of the intervention and reported that it sometimes felt unnatural.

*“…we need to follow these questions and we need to do it this way, but that’s not real life and that’s not how we would speak to our ordinary patients that don’t have a mental health illness… you had to follow this stream of questioning, and that didn’t work…It wasn’t comfortable because that’s not the normal of working….”* (Staff 5, Nurse, 40’s)

#### Relational integration

Relationships between practice staff and staff delivering the intervention and patients were considered as important in the implementation of the intervention. The availability of team support within some practices facilitated intervention delivery, whilst the lack of availability hindered progress. A positive relationship between patient and staff members encouraged confidence and trust in staff members to deliver the intervention.

##### The level of ‘in-house’ support

Staff suggested that they required support from team members within practices to deliver the intervention. There was a need for access to health advice for difficult cases when staff were unsure. However, there was a variation between the level of teamwork within different practices to facilitate intervention delivery. This sometimes acted as a barrier to providing the intervention, particularly in cases where permission was required for prescribing medications and senior staff members were unavailable.

*“I did feel as if I was on my own a little bit in the surgery… There was just not support as in, I’m worried about this patient, but it was maybe just reading consultation notes, that sort of thing.”* (Staff 10, Nurse, 40’s)

The differences between team working across different practices were apparent when other staff reported that senior members within their practice were willing to provide advice regarding patients, which facilitated intervention delivery.

*“I’ve always got backup. I wouldn’t have hesitation in asking any of the senior and the qualified staff. I think that we’ve got the backup here to do a really good job”* (Staff 14, Nurse, 50’s)

##### Patient – staff alliance

It was apparent that some patients and staff had formed close therapeutic relationships. Staff were aware that in order to ensure that patients felt comfortable engaging with the intervention, and instil confidence in their ability of providing it, it was important to establish connections with patients.

*“Making them feel comfortable by making a relationship with them to start with… And that is making a relationship with them to come back and encouraging them in their own way.”* (Staff 11, Nurse, 60’s)

As a result of the relationships patients had formed with staff, most patients felt positive about interacting with staff and found that this increased their willingness to engage with the intervention.

*“…we had quite a good relationship, she’s very supportive and I think she understood about me personally, obviously having my medical record, that it was the medication that stopped me losing weight ....”* (Patient 12, female, 70’s)

#### Skill set workability

Staff knowledge regarding mental health were both barriers and facilitators to intervention delivery. Most staff appeared knowledgeable regarding physical health. Once staff had received the PRIMROSE study intervention training, it was clear that they developed valuable skills that aided intervention delivery.

##### Knowledge

Staff reported a lack of experience working with patients with mental health problems resulting in a lack of knowledge in this area. As a result, they felt anxious about delivering the intervention. One staff member suggested that the intervention was not in keeping with her knowledge and therefore it would be more appropriate that patients were seen in mental health settings.

*“I just think better in a mental health environment, with the nurses that already have that knowledge of conditions. Because they were very limited on what knowledge we do know about mental health.”* (Staff 13, Nurse, 30’s).

This theme was atypical among patients but demonstrated that patients had noticed that staff appeared to be lacking in confidence providing the intervention.

*“… she was kind of paddling in the dark to some degree… the impression I got is that, you know, she was just given a pile of information and she had to do her best to interpret…”* (Patient 81, male, 60’s)

In contrast, staff with prior experience with mental health patients were more knowledgeable about interacting with people with mental health and less anxious delivering the intervention. In some cases, this prior experience facilitated their knowledge on delivering the intervention to this population.

*“Maybe I was using the tools and the skills I used for really poorly mentally ill patients that were having to be put on a ward for their own safety.”* (Staff 14, Nurse, 50’s)

Whilst there was a mixture of staff that had knowledge of mental health, it was clear that most staff had some form of knowledge of physical health as a result of their prior experience.

*“… I do the NHS health checks here, if their risk is high and their total cholesterol is high or their ratio is high, I will actually go and initiate a start on the total statin 20mg…if the patient was coming to me wanting to lower their cholesterol or lower their blood pressure, then I have the tools that I can, advise them on that”* (Staff 14, Nurse, 50’s)

##### Training

Staff reported thatthe training delivered prior to the intervention was essential in increasing their knowledge of how the intervention could be delivered. In most cases staff reported that the training increased their confidence and prepared them in terms of how to engage with people with SMI.

*“…the training you gave us was amazing, very helpful, I wouldn’t have been able to do it without it, just as a background of the different illnesses and going back to basics for us…And also we had some training on communication and body language and things like that, which was a good refresher, because you forget.”* (Staff 2, HCA, 50’s)

However, not all patients believed that staff had been trained adequately. One patient felt that HCAs had less training and were consequently unable to deliver the intervention and staff commonly suggested further training would be beneficial.

*“We had to use Health Care Assistants, who maybe didn’t have quite as much training…”* (Patient 17, male, 40’s)

*“… it seemed to sort of suit what we had to do but, like you say, perhaps something in the interim would be good”* (Staff 12, HCA, 30’s)

##### Skills

Patients reported on the skills that staff members used when delivering the intervention. It was evident that staff provided advice when necessary, displayed patience when interacting with patients, were clear, encouraging and positive.

*“…sometimes I wasn’t sure, but then <practice nurse> would kind of give me a bit of hand with ideas, and then we’d kind of come up with it together…it was good….”* (Patient 6, female, 30’s)

This was in some cases supported in staff accounts. Staff commonly reported attempts to make appointments engaging by interacting with patients and providing guidance when required. This demonstrated staff interpersonal skills.

*“…when a patient comes in, I try to make it as much fun as possible as well, because doing that... it makes it more comfortable for the patient, I think.”* (Staff 1, HCA, 40’s)

#### Contextual integration

The integration of the intervention into practice contexts contained challenges., Resources including time were required to successfully implement the intervention and the availability and time taken to identify this in some cases acted as a barrier to delivering the intervention.

##### Resource availability and benefits

Staff described that one of the main intervention functions involved searching for available local services that patients could be referred to for additional support in reducing physical health problems. However, one of the barriers to implementing the intervention was finding the time required to look for such resources as well as the lack of availability of local services.

*“…part of the Primrose it was to look what was available in the area, and to be perfectly honest I didn’t have time. We did some of it but you just don’t… you haven’t always got time to sit and read through a directory of things and see what’s around in the city.”* (Staff 5, Nurse, 40’s)

The need for external resources to implement the intervention was highlighted where staff had successfully located resources. They suggested that this facilitated easier access to resources which appeared to help patients achieve specific health goals.

*“Another person was referred to a dietician. As I said, that was in regard to his cholesterol, and he was referred to a specialist, as well. And I think that probably happened a bit more quickly than it might have done… I think we’re quite lucky, out here, that you can refer people on different groups, clubs…”* (Staff 4, HCA, 50’s)

### Reflexive monitoring

When evaluating ways to adapt the intervention, it was suggested that there was a need for a PRIMROSE designated clinic within future practices and technology to make the intervention more workable.

#### Modifiability through accessibility

It is also been reported within interactional workability (accessibility) that staff made necessary adjustments to fit intervention appointments into routine practice in settings where time was limited. Some staff suggested that a designated timeslot could be developed for patients within GP surgeries to better integrate the intervention and allow more time for appointments.

*“…rather than have the appointments scattered, have an actual little clinic for it, and then let, let people know what day that you’re running and to come in…”* (Staff 1, HCA, 40’s)

#### Resource availability and benefits

Some patients suggested that there was a need for current digital technology in order to track their progress during the intervention. However, the intervention did not facilitate access to these types of tools, and GP practices in routine settings do not have access to such facilities.

*“I have an apple iPhone and I track my weight and my BMI and my measurements on my phone. And I would have liked something like that could have tracked it, not necessarily that it was always on paper.”* (Patient 17, male, 40’s)

## Discussion

This was one of the first qualitative studies to explore the implementation of a CVD-risk reducing intervention into primary care using the NPT. Staff and patients both reported several barriers and facilitators to implementing PRIMROSE into primary care, which are important for the implementation of future interventions of this kind for people with SMI in primary care. A common finding in the literature is that mental health stigma negatively impacts the delivery and implementation of physical healthcare to patients with SMI [12, 15]. We also found that mental health stigma influenced cognitive participation and therefore staff willingness to deliver PRIMROSE (i.e. cognitive participation), which subsequently influenced confidence to deliver the intervention. This appeared to stem from skill-set workability and a lack of knowledge in mental health. This suggests that more work is needed to instil confidence in primary care professionals in terms of engaging with people with SMI and tackling stigma. This notion was further supported where some staff reported that prior experience and knowledge in mental health meant that they were more willing to deliver the intervention, and PRIMROSE training further increased confidence. Thus, mental health specific training for primary care professionals (practice nurses and health care professionals) could help to facilitate the implementation of physical health interventions in people with SMI. Recent work supports the value of training primary care professionals in mental health, with findings suggesting improvements to knowledge, attitudes and practice [23]. We also found that skill-set workability in terms of physical health knowledge played an important role in facilitating implementation, which mirrored previous findings, however patients in the present study thought that HCAs had less physical health knowledge than nurses [13–15]. This may be explained by the fact that HCAs training in the UK covers basic nursing skills and involves obtaining the Care Certificates (level 2 Certificate in Healthcare Support Services and level 3 Diploma in Healthcare support) which can be earned through apprenticeships. HCA training is much shorter than nurses who are educated to degree level. Therefore, training of primary care teams in both physical and mental health-specific knowledge could facilitate the implementation of physical health interventions in people with SMI but HCAs may require more training than nurses to deliver this type of intervention.

Previous work reported that patients with SMI find it difficult to access appointments in primary care, consequently affecting the uptake of physical health interventions [7, 15]. In the present study, problems related to interactional workability influenced collective action, where despite staff efforts to increase accessibility to appointments, there was a perceived lack of patient engagement with the intervention, which subsequently negatively impacted implementation. This was consistent with previous work that suggested that lack of engagement with appointments impeded physical health intervention delivery [15]. Other reasons for lack of patient engagement despite improve scheduling were not discussed in interviews. However, this lack of engagement was reported from the perspective of staff and patients that were interviewed did not report this. This is possibly due to the notion that this sub-sample were more engaged with the intervention as they mostly attended 6 or more sessions.

It was previously reported that a lack of interpersonal skills displayed by HCPs and lack of continuity of care between different HCPs impeded access to and implementation of physical health interventions for people with SMI [6, 7, 14, 15]. We also found that relational integration, in terms of the relationships formed between staff and patients was particularly integral to the implementation of the PRIMROSE intervention. There was a continuity of care for those that did attend appointments frequently which subsequently facilitated positive relationships with staff. It is possible that one-to-one contact with the same HCP enabled patients to feel more comfortable, more trusting of primary care professionals and therefore more likely to engage with the intervention. The value of continuity of care for people with SMI is supported in other work where ongoing personal relationships with family, friends and different healthcare professionals facilitate trust and are central for mental health recovery [24, 25]. This may explain findings from the trial that the PRIMROSE intervention was associated with lower costs due to reduced psychiatric admissions [16].

A common finding was that staff questioned the contextual integration of the intervention; most staff did not perceive the intervention as applicable to real-world practice, because there was a lack of access to resources, time, and relational integration in terms of staff support required for implementation into primary care. These findings are well cited in previous work where the lack of availability of services, appointment waiting times, staffing levels and challenges surrounding increased workload affect the delivery and implementation of physical healthcare for people with SMI [12–14]. Conversely, we found that when staff did have access to specialist resources and designated PRIMROSE appointment slots, this facilitated intervention delivery. In fact, staff and patients engaged in reflexive monitoring and suggested the intervention could be further developed including having a designated timeslot However, primary care is often an over-stretched service and if interventions of this kind are to be implemented in such settings, more consideration must be given to the resources required to facilitate successful delivery.

### Implications for practice

To summarise, our findings suggest that training in physical health, mental health knowledge, positive relationships with patients, designated clinics in primary care and extra resources including additional staff are important to facilitate implementation. We therefore recommend that the following should be taken into account when implementing physical health interventions specific to reducing CVD risk and/or more broadly related to health promotion for people with SMI in primary care: a) training should cover physical and mental health, b) application of interpersonal skills when engaging with patients, and access to the same HCPs to maximise continuity and trust and c) increasing accessibility to resources including specialist services, more staff and designated time-slots for people with SMI in primary care settings.

### Limitations and strengths

Overall, the sample that took part in the current study captured the variation of characteristics of both staff and patients that took part in the PRIMROSE study, in terms of age, gender (patients), years of experience (staff) and mental health diagnosis (patients). It is however likely that participants may have been more engaged with the intervention as the majority of the sample attended and delivered many appointments. It is possible that less engaged participants may have expressed opposing views. The views of participants from different ethnic groups (both groups) and gender (staff) may have also not been captured adequately. Staff and patients were also interviewed sometime after the last intervention appointment (an average of 8 months in staff and 11 in patients). Staff were however, in most cases able to recall events related to PRIMROSE as reflected in the rich data that was captured.

Further, the researchers (S.H2, A.B) had prior involvement in PRIMROSE and prior contact with staff delivering PRIMROSE as well as conducted the present staff interviews and coded transcripts. It is possible that researchers’ pre-conceptions of staff and knowledge about PRIMROSE may have influenced their interpretation of findings as well as the way in which data were collected. For example, social desirability and/or previous negative experiences between researchers and staff may have played a role. However, we minimised this by discussing themes with the wider team, explaining to staff and patients that both negative and positive views were encouraged and involved two other researchers (S.H2 & J.R) in the interpretation of data not involved in the PRIMROSE trial or data collection.

Finally, a further strength is that the use of the NPT helped to further explain factors related to successful and unsuccessful implementation in primary care. We applied the NPT using an inductive data-driven approach, whereby themes were identified in the raw data first and then mapped to NPT constructs. We also utilised the expertise of an implementation scientist (J.R) as well the expertise of the wider team to interpret the NPT in relation to themes accurately.

## Conclusions

To our knowledge, this is one of the first studies to explore the implementation of a behavioural physical health intervention delivered by primary care staff to patients with SMI in primary care practices using NPT. Successful implementation hinged on staff preconceptions, experience and knowledge of mental health, training, physical health knowledge, continuity of care and relationships between staff and patients, interpersonal skills displayed by staff and contextual factors including resource. Our findings should be used as basis for informing the implementation of future interventions into primary care settings.

## Supplementary information

**Additional file 1.**

## Data Availability

The data are not publicly available as they contain confidential information.

## Abbreviations

CVD: Cardiovascular disease
SMI: Severe mental illness
HCA: Healthcare assistant
HCP: Healthcare professional
NPT: Normalisation process theory
UK: United Kingdom
PRIMROSE: Prediction and management of cardiovascular disease risk for people with severe mental illnesses: A research programme and trial in primary care
GP: General Practitioner
