# Supplementary material for: A qualitative study exploring the barriers and facilitators of implementing a cardiovascular disease risk reducing intervention for people with severe mental illness into primary care contexts across England: the ‘PRIMROSE’ trial
Source: BMC Health Serv Res. 2020 Aug 15;20:753. doi: 10.1186/s12913-020-05643-2 (PMC7429749; doi:10.1186/s12913-020-05643-2)
Supplement: Supplementary file 1 — Additional file 1. [file 12913_2020_5643_MOESM1_ESM.docx]

**PRIMROSE Intervention characteristics**

| **Brief name** | A primary-care led intervention to reduce CVD risk in people with SMI through health promotion. |
| --- | --- |
| **Why** | To reduce cardiovascular disease risk in people with SMI through health promotion and physical health medication. |
| **What** | - Nurses and HCAs were given a manual to follow in sessions comprising of eight behavioural strategies. - Goal setting (e.g taking statin medications, increasing physical activity etc.), making an action plan (e.g. a step by step plan of how to achieve a goal), recording progress with goals (e.g. recording reduction in weight), providing positive feedback (e.g. displaying praise when achieving successes), involving supportive others (e.g. encouraging involvement from friends and/or family by carrying out healthy behaviours), reviewing progress (e.g. discussion of progress with goals), coping with setbacks (e.g. discussing how to overcome potential problems) and habit formation ( e.g. repeating behaviours in specific contexts). - Training was conducted by a practice nurse with expertise in mental health, a health psychologist, a lived experience trainer and program manager. - Training was held over two days and covered the link between CVD and SMI, mental health, interventions to lower CVD risk, behaviour change techniques, and strategies to encourage motivation. - The manual included a step by step appointment flow chart, how to manage CVD risk and facilitate patient motivation. - Patients were given health plans to record their goals and monitor their behaviour. - Part of the intervention also involved signposting patients to specialised services available in the area if necessary such-as smoking cessation programmes. |
| **Who provided** | - Nurses and HCAs were the usual staff within the practices and were trained and up-skilled to deliver the intervention. - Their usual role involved monitoring patients within practices and carrying out routine health checks but not providing care specifically for mental health. |
| **How** | - Face-to-face where possible or on the telephone |
| **Where** | - The setting was UK primary care. |
| **When and how much** | - The intervention was delivered across 8-12 sessions over 6 months - Sessions lasted approximately one hour for the first and final appointments and 20-30 minutes for every other appointment. |
| **Tailoring** | - Health promotion goals were set based on the needs of patients and agreed upon with patients. |

**Healthcare professional Topic Guide Questions**

**1. Introduction (5 mins)**

*a) Nature and purpose of research*

The aim of this study is to explore your experiences of delivering the PRIMROSE intervention to patients with SMI who are at a high risk of developing CVD. We are also interested in your views on the training provided to help you deliver the intervention.

We would like to know what you think the barriers and facilitators were to delivering the intervention and your thoughts on whether or not the training equipped you with the right skills for delivering the intervention

We are interested in all of your thoughts today so please share any positive or negative views on the primrose intervention and training programme.

Do you have any immediate thoughts/comments or questions before we start?

*b)* *Anonymity of presentation of results*

*• Introduce digital audio recorder*

*• Stress confidentiality*

*• Set ground rules*

*• Advise that there are no right or wrong answers*

*• Discuss length of interview*

*• •Sign consent form*

**2. Introduce participants & context. Start recording**

a) Ask each participant to say a little bit about themselves*:*

- *What previous experience of research have you had?*
- *Why did you agree to take part in PRIMROSE?*

**3a) Intervention Experience – Impact on nurses/HCA**

1. Can you start by telling me a little about your experiences of the PRIMROSE study?

(*How have you found it*?)

1. Can you tell me about/describe a typical intervention appointment for you? What did you do with the patient? Can you give me an example of that?
2. Can you tell me about an intervention appointment that you felt went well?

- *What do you think went well?*
- *What do you think could have been done differently?*

1. Can you tell me about an intervention appointment which was more difficult for you?

- *Why do you think this didn’t go so well?*
- *What do you think could have been done differently?*

e) What do you think about the service in general?

*- What has gone well?*

*- What has gone badly/not so well?*

*- Is there anything you would change?*

- *e.g. frequency of appointments, length, how many?*

**3b) Intervention Experience – impact on patients**

a) Can you give me an example of a patient who made progress?

- *Why do you think this occurred?*
- *How did you manage this?*
- *Is there anything with hindsight you might do differently with this patient?*
- *Can you think of anything else that would make a difference?*

b) Can you give me an example of a patient who made little/no progress or got worse?

- *Why do you think this occurred?*
- *How did you manage this?*
- *Is there anything with hindsight you might do differently with this patient?*
- *Can you think of anything else that would make a difference?*

c) What factors do you think might have kept the patients motivated to see you?

- *For example, were there supportive others who encouraged the patient to attend, the relationship you had with the patient etc.*

d) How did you encourage patients to attend?

e) What factors might have caused a patient not to attend the intervention appointments?

- *For example, time of appointments, personal situation?*

f) In general how do you think the intervention has had an impact on patients?

- *Can you give me an example? Was there anything tangible that you could see as evidence of impact on mental health and physical health? Explore positive and negative*

g) Do you think it would be possible to offer this intervention to patients as part of regular practice? If yes then how, if no then why not?

**3c) Intervention experience**

a) What previous experience have you had with patients with SMI (Formal/Informal?)

*- How did you feel about working with this particular patient group?*

b) What do you think about your skills to deliver the intervention?

- *Mental health*
- *Physical health*
- *Medications*

c) What did you think about the training? (if helpful then how, if unhelpful then why not)

- *If not, what was needed?*
- *Anything you would change/alter/improve?*
- *Practical aspects such as location, timing etc, manuals, material, trainers*

**B. Professional roles and identity**

a) How do you see the work you did in PRIMROSE fitting in your normal job role?

*- Explore why it did or didn’t fit*

*- Explore whose role it might be if they didn’t feel like it fit*

**C. Beliefs about consequences**

1. Reflecting on your experience what do you think the benefits/advantages might be of the intervention?

- *for patients/you/the practice/other practice staff*

1. What do you think might be the disadvantages?

- *for patients/you/the practice/other practice staff*

**D. Memory, attention, decision processes**

a) Were there any circumstances where you decided not to deliver the intervention to a patient?

- *Particular appointments or entire intervention*
- *Particular patients?*
- *Reasons why?*

**E. Environment and resources**

a) What resources did you need to help you carry out the intervention?

- *Other people covering your normal duties? Admin support?*
- *Access to support from team, colleagues, GP, other PRIMROSE nurses?*

b) What influenced your ability to provide the intervention?

- *(resources, needs of patient/ other patients, availability of equipment/space, time, competing tasks,* *practice team’s views/attitudes)*

c) How did you plan intervention appointments?

- *Prompts/reminders for invitation?*
- *How was this information recorded and monitored?*
- *Draw upon experiences in other long term condition management – e.g. diabetes.*

**F. Behavioural regulation**

a) How did you monitor attendance?

**G. Emotions**

a) How did you feel about delivering the intervention?

- *Confidence?*
- *What emotional response did it evoke? Fear, anxiety etc? Why?*
- *If negative emotion, what do you think might have helped you to change this response?*

**6. Finishing the Intervention**

1. How did you feel about ending the intervention & finishing the sessions with patients?

- *What did your patients feel about it coming to an end?*
- *What aspects were discussed at the final appointment?*

**7. Going Forward**

1. How will you use the training/experience gained from the PRIMROSE study going forward in regular practice?
2. If you were going to continue to provide the intervention how would you suggest it be done in your practice?

- *Should anything be changed?*
- *Length of appointments*
- *Frequency of appointment*
- *Support provided*
- *Training provided*

1. What would be your thoughts on providing this intervention long term?

**7. Conclude interview**

Do you have any other comments to make regarding your experiences as a PRIMROSE intervention nurse?

I just want to finish by saying thank you for being a part of PRIMROSE and thank you for taking part in this interview.

**Patient Topic Guide Questions**

**1. Introduction**

*a) Nature and purpose of research*

Thank you very much for taking part in the interview today, we really appreciate you giving your time to talk to us. Please be honest and open about your views, there are no right or wrong answers so any thoughts or comments you have please do share them.

We want to explore your experiences and views on the PRIMROSE service that you have recently received at your GP practice.

Do you have any immediate thoughts/comments or questions before we start?

b) Anonymity of presentation of results

- Introduce digital audio recorder
- Stress confidentiality

**2. Experience of receiving the service**

a) Why did you agree to take part in the PRIMROSE service?

b) Were you able to access the nurse/HCA to take part in the PRIMROSE service?

*- how were you contacted?/ any difficulties?/anything that helped make accessing the service easier?*

c) Did you ever talk on the telephone vs. meeting in person?

*- What was good/bad about these telephone sessions? Why?*

d) Did you understand the purpose of the PRIMROSE service appointments?

*- Ask the patient to summarise the purpose?/Who explained things to you?/How was information given?/Could it have been explained better?*

e) Were you able to set a goal that was relevant to you during your appointment with the nurse/HCA?

f) Is there anything you did not like about the PRIMROSE service?

g) Was there anything good about the PRIMROSE service provided by the nurse/HCA?

h) Did you find the PRIMROSE appointments useful?

*Why?/What in particular?*

i) Do you think the service has had a positive effect on your physical health?

*- In what way? Can you give me an example?*

j) Do you think that anything could be improved about the PRIMROSE service?

*- What? How? What services would you like to receive to lower your risk of CVD?*

k) What might have helped you to take part in the PRIMROSE service*?*

*- What might make it easier?/What might make it difficult?*

l) Would you recommend this type of service to someone else?

*- If yes, why?/If not, why not?*

m) How does the PRIMROSE service compare with the usual care you receive from your GP practice?

**3. Social Support**

a) Were you given the opportunity to involve anyone in the primrose service?

- *How was this discussed with you?*
- *How was it suggested they were involved?*
- *Were you given a choice over how they were involved?*

b) How they were involved?

c) How would you have liked this person to be involved?

d) When thinking about your experiences with your GP practice – has the involvement of supportive others been discussed with you before?

*Yes – how was this explored with you?*

*Different in different services?*

*No – would you like this to be explored? What would you like to see happen?*

e) When thinking about your experiences with other health services – has the involvement of supportive others been discussed with you before?

*Yes – how was this explored with you?*

*Different in different services?*

*No – would you like this to be explored? What would you like to see happen?*

*How does this compare with their experience in primary care?*

f) What might have helped you to involve someone in the PRIMROSE service*?*

*- What might make it easier?*

*- What might make it difficult?*

g) How do you think that the involvement of someone in your care could be improved?

**8. Conclude discussion**

I just want to finish by asking you:

- Is there anything that has not been discussed that you would like to raise?
- Are there any other comments/questions or ideas?
